# Role of Site-Specific Glycosylation in the I-Like Domain of Integrin β1 in Small Extracellular Vesicle-Mediated Malignant Behavior and FAK Activation

**DOI:** 10.3390/ijms22041770

**Published:** 2021-02-10

**Authors:** Lin Cao, Yurong Wu, Xiuxiu Wang, Xiang Li, Zengqi Tan, Feng Guan

**Affiliations:** Joint International Research Laboratory of Glycobiology and Medicinal Chemistry, College of Life Science, Northwest University, Xi’an 710069, China; lincao@stumail.nwu.edu.cn (L.C.); wuyurong@stumail.nwu.edu.cn (Y.W.); 18829270128@163.com (X.W.); xiangli@nwu.edu.cn (X.L.); zengqtan@nwu.edu.cn (Z.T.)

**Keywords:** integrin β1, N-glycosylation, sEVs, FAK, migration, adhesion

## Abstract

Integrin β1 plays an essential role in the crosstalk between tumor cells and their microenvironment. Aberrant N-glycosylation of integrin β1 was documented to alter integrin β1 expression, dimerization, and biological function. However, the biological function of site-specific N-glycosylation of integrin β1 on extracellular vesicles is not fully understood. In this study, we mutated putative N-glycosylation sites in different domains of integrin β1. Removal of the N-glycosylation sites on the I-like domain of integrin β1 (termed the Δ4–6 β1 mutant) suppressed focal adhesion kinase (FAK) signaling, cell migration, and adhesion compared with other β1 mutants. Cell adhesion, migration, and activation of FAK were suppressed in recipient MCF7 cells co-cultured with Δ4–6 mutant cells and treated with small extracellular vesicles (sEVs) from Δ4–6 mutant cells. Notably, the wild-type and β1 mutant were both present in sEVs, and could be transferred to recipient cells via sEVs, resulting in changes of cell behavior. Our findings demonstrate the important roles of N-glycosylation of the I-like domain of integrin β1. Moreover, the vesicular Δ4–6 β1 mutant can regulate integrin-mediated functions in recipient cells via sEVs.

## 1. Introduction

Small extracellular vesicles (sEVs), one type of membrane-covered structure (diameter 30–100 nm), originate from the endosomal pathway, are secreted by exocytosis into the surrounding extracellular space, and are present in various body fluids [1,2]. sEVs carry plentiful bioactive materials, including proteins, nuclear acids, and lipids, which mediate various types of cell-to-cell communication [3]. Tumor-derived sEVs could promote proliferation, migratory ability, and transfer chemoresistance and modulate immune response through release of embedded molecules into recipient cells [4,5]. Recent studies have documented that vesicular epidermal growth factor receptor (vEGFR) from gastric cancer cells would be transferred into liver stromal cells, reprogramming the liver microenvironment and promoting cancer cells’ liver-targeted metastasis [6]. Several studies have confirmed the intercellular transfer of integrin by sEVs to modulate the physiology of recipient cells [1,7]. For example, vesicular integrins determine the organ-specific metastasis of derived tumor cells, and blockage of integrin binding with extracellular matrix (ECM) was found to decrease sEV uptake and tumor metastasis [7].

Integrin, a typical transmembrane receptor, mediates cytoskeleton remodeling and intracellular signaling pathways’ activation by binding with ECM components [8]. There are 18 α and 8 β subunits consisting of 24 intact integrins. Especially, integrin β1, of the β subfamily, is able to bind with each of 12 α subunits to form different types of heterodimers. Recent studies have documented that aberrant expression of integrin β1 and dysregulation of focal adhesion kinase (FAK)/protein kinase B (AKT) signaling closely correlate with the malignant phenotype [9,10]. For example, cell migration and invasion were enhanced by gamma-synuclein through the activation of the integrin β1–FAK signal pathway in colorectal cancer cells [11]. Specifically, integrin β1 was reported to be glycosylated. Aberrant glycosylation of integrin β1 affected its biological function and altered cell adhesion, migration, and survival in cancer [12]. Isaji et al. elucidated that removal of N-glycosylation sites on the I-like domain of integrin β1 significantly suppressed β1 expression and heterodimeric formation, leading to the inhibition of cell spreading [13]. Hou et al. revealed that membrane-proximal N-glycosylation on integrin β1 could regulate cell migration by promoting integrin β1 activation [14]. In hela cells, knockdown of O-GlcNAc transferase increased the focal adhesion complex formation of integrin β1 and FAK, as well as levels of active integrin β1, resulting in the promotion of cell adhesion and suppression of cell migration [15].

Previous study revealed the biological function of N-glycosylation in different domains of integrin β1 [13,14]. Our recent study revealed that the migration ability of recipient cells was suppressed by vesicular integrin β1 with high levels of bisecting GlcNAc modification [1]. However, the effect of N-glycosylation in different domains of integrin β1 on biological function of sEVs is not well elucidated. In the present study, we validated the function of N-glycosylation in different domains of integrin β1 in donor MDA-MB-231 cells by sequential site-directed mutagenesis. Moreover, we describe the manner in which various β1 mutants modulate the function of sEVs and the behaviors of recipient cells.

## 2. Results

### 2.1. N-Glycosylation Site Mutation of Integrin β1

**Figure 1:**
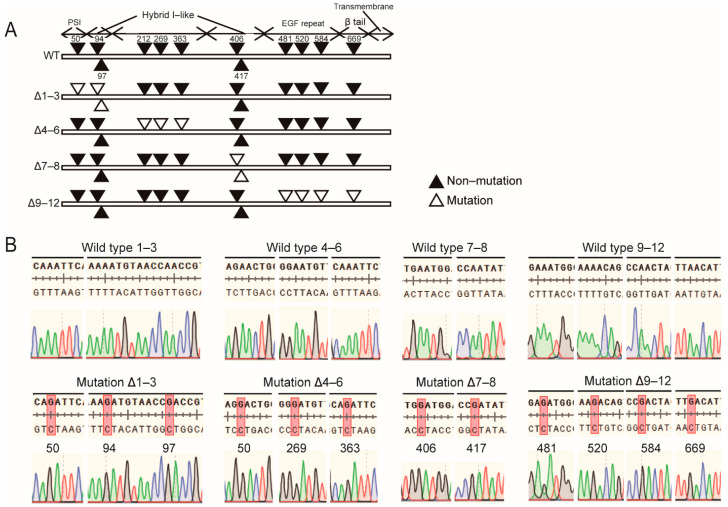
Wild-type and N-glycosylation mutants of integrin β1. (**A**) Potential N-glycosylation sites of integrin β1. Closed triangles represent N-glycosylation, and open triangles represent point mutations. (**B**) DNA sequences of β1 mutants constructed by site-specific mutagenesis.

Previous study had illustrated that 12 N-glycosylation sites characterized by asparagine residues within the NX(S/T) consensus sequence exist on integrin β1. Among them, the N564 and N74 sites do not normally carry N-glycan [16]. The other 10 N-glycosylation sites were respectively located in the plexin-semaphorin-integrin (PSI) domain, integrin-epidermal growth factor domain (I-EGF), I-like domain, and β-tail domain (Figure 1A). To investigate the effect of losing N-glycan modification at given sites on the expression and biological function of integrin β1, we converted 10 asparagine residues within the above four domains into aspartic acid through site-specific mutagenesis. The primers used for amplification and mutation of integrin β1 are shown in Table 1. The constructed mutants are listed below: the Δ1–3 β1 mutant in PSI and upstream of the hybrid domain, Δ4–6 in the I-like domain, Δ7–8 downstream of the hybrid domain, and Δ9–12 in the I-EGF domain (Figure 1A). The mutant base sequence is listed in Figure 1B. 

### 2.2. Influence of Integrin β1 Mutants on the Cell Adhesion, Mobility, and Signaling Pathway

**Figure 2:**
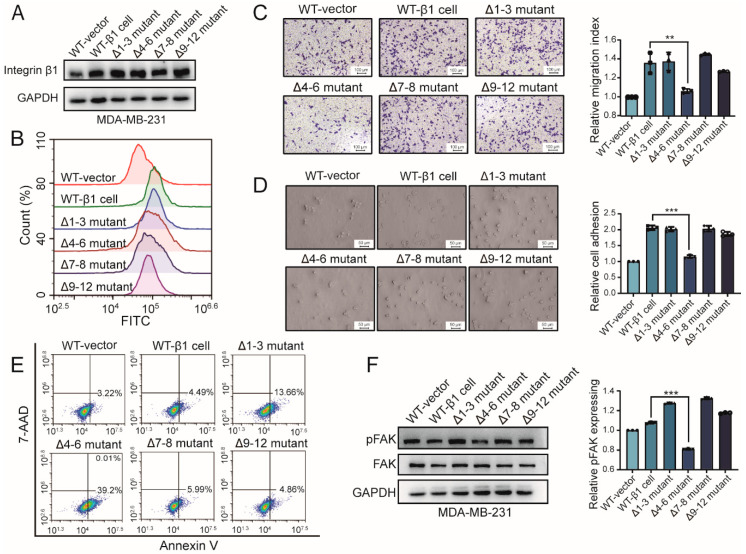
Effects of β1 mutants on behavior of MDA-MB-231 cells. (**A**) Expression of integrin β1 in constructed mutant cells by Western blotting. (**B**) Surface expression of integrin β1 in constructed mutant cells by flow cytometry. (**C**) Cell adhesion on fibronectin (FN) in wild-type (WT) and mutant cells. ** *p* < 0.01. (**D**) Migration of WT and mutant cells by transwell assay. *** *p* < 0.001. (**E**) Apoptosis of WT and mutant cells by flow cytometry (**F**) Focal adhesion kinase (FAK) activation of WT and mutant cells. *** *p* < 0.001. Full-length blots/gels for Figure 2A,F are shown as Figure S1.

N-glycosylation plays numerous roles in protein folding, dimerization, turnover, and so on. To explore the biological function of the site-specific N-glycosylation of integrin β1, wild-type (WT) β1 and constructed β1 mutants were introduced into MDA-MB-231 cells, termed wt-β1 cell, Δ1–3 mutant, Δ4–6 mutant, Δ7–8 mutant, and Δ9–12 mutant, respectively. The expression of integrin β1 in β1 transfectants was elevated compared with MDA-MB-231 cells tranfected with empty vectors. There was no significant difference in amounts of integrin β1 between different transfectants, as shown in Figure 2A. Moreover, flow cytometry analysis revealed an increased expression of β1 integrin on the cell surface of wild-type β1 transfected and mutant cells, compared with those of wild-type MDA-MB-231 cells, shown as Figure 2B. These results suggest that the N-glycosylation of integrin β1 had a rare influence on its expression in MDA-MB-231 cells, which was consistent with a previous study [14]. We wondered whether N-glycosylation in different domains of integrin β1 affected behaviors of MDA-MB-231 cells. Although the expression of integrin β1 was comparable to that of WT cells, cell migration and adhesion on fibronectin was significantly decreased in the Δ4–6 mutant compared with other mutants (Figure 2C,D). A previous study reported that overexpression of integrin β1 confers resistance to apoptosis in hepatoma cells [17]. Our flow cytometry analysis revealed increased apoptosis in Δ1–3 and Δ4–6 mutants rather than other mutants, as shown in Figure 2E, which demonstrated that N-glycosylation in the I-like domain is essential for integrin-β1-mediated apoptosis resistance. Activation of FAK is an important step for integrin-β1-mediated signaling [18,19]. Our Western blotting analysis showed that response to the fibronectin (FN)-induced activation of FAK was attenuated in the Δ4–6 mutant compared with other mutants (Figure 2F). Collectively, these data indicate that N-glycosylation in the I-like domain might be involved in regulation of various cellular functions via the FAK signaling pathway.

### 2.3. Effects of Secreted Components from β1 Mutants on Behavior of Recipient Cells

**Figure 3:**
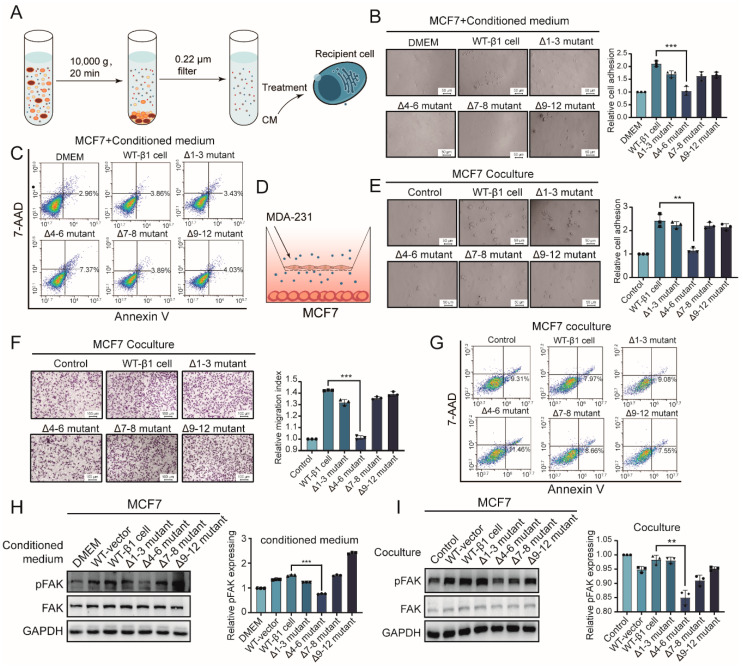
Effects of secreted components from β1 mutants on behavior of recipient cells. (**A**) Workflow of conditioned medium (CM) preparation and treatment. (**B**) Adhesion ability on FN of MCF7 cells treated with CM from mutant cells. *** *p* < 0.001. (**C**) Apoptosis of MCF7 cells treated with CM from β1 mutants by flow cytometry. (**D**) Workflow of the co-culture system. (**E**) Adhesion of MCF7 cells on FN co-cultured with β1 mutants. ** *p* < 0.01. (**F**) Migratory ability of MCF7 cells co-cultured with β1 mutants by transwell assay. *** *p* < 0.001. (**G**) Apoptosis of MCF7 cells co-cultured with β1 mutants by flow cytometry. (**H**,**I**) FAK activation of MCF7 cells treated with CM (**H**) or co-cultured (**I**) with β1 mutants by Western blotting. Results are presented as mean ± SD from three independent experiments. ** *p* < 0.01, *** *p* < 0.001. Full-length blots/gels for Figure 3H,I are shown as Figure S2.

It was documented that less-malignant tumor cells display enhanced migratory behavior by interaction with malignant tumor cells [20]. We were interested in observing if secreted components from mutants could affect the behavior of less-malignant recipient MCF7 cells. Conditioned medium (CM) from the above-described mutants was prepared (Figure 3A). We treated MCF7 cells with CM, which showed less malignant behavior than MDA-MB-231 cells. As shown in Figure 3B, cell adhesion of MCF7 cells was suppressed by Δ4–6-CM but enhanced by CM from other mutants. Flow cytometry analysis suggested increased apoptosis of MCF7 cells treated by Δ4–6-CM (Figure 3C). Consistently, experiments using a co-culture system (Figure 3D) showed that adhesion and migration of MCF7 cells were inhibited by soluble compounds released by the Δ4–6 mutant but not by others (Figure 3E,F). Similarly, co-incubation with the Δ4–6 mutant suppressed apoptosis of MCF7 cells (Figure 3G). Mechanically, activation of FAK in MCF7 cells was suppressed by CM and co-incubation with the Δ4–6 mutant but enhanced by that of other mutants (Figure 3H,I).

Together, these results indicate that the behaviors of recipient cells could be influenced by soluble compounds released from integrin-β1-transfected cells. N-glycosylation in the I-like domain is essential for the biological function of integrin β1 from soluble compounds.

### 2.4. Effects of β1 Mutants on Biological Function of sEVs

**Figure 4:**
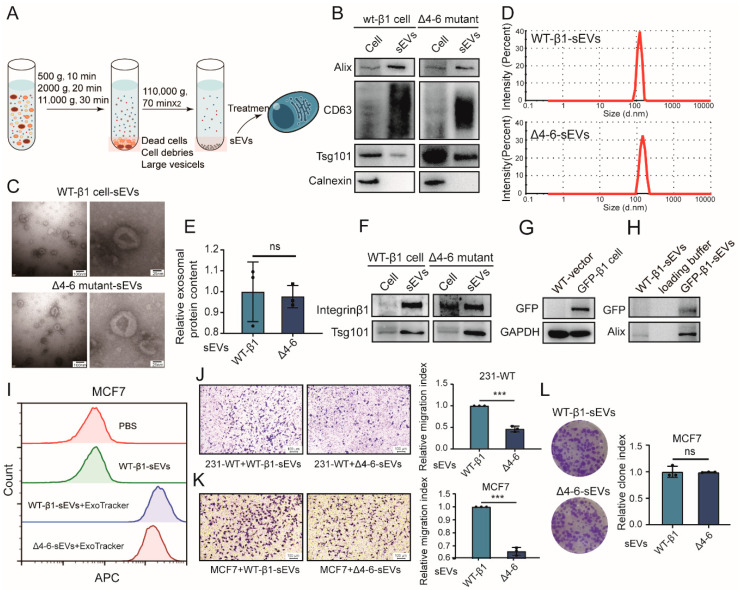
Effects of β1 mutants on the biological function of small extracellular vesicles (sEVs). (**A**) Workflow of sEV isolation. (**B**) Expression of sEV markers by Western blotting. Corresponding donor cell proteins and 10 μg of sEVs were loaded. (**C**) Morphology of sEVs by TEM. (**D**) Particle size of sEVs from WT or Δ4–6 mutant cells by nanoparticle tracking analysis (NTA). (**E**) Secretion of sEVs quantified by protein concentration using a bicinchoninic acid (BCA) reagent kit. ns, not significant. (**F**) Expression of integrin β1 on sEVs from WT or Δ4–6 mutant cells by Western blotting. (**G**) Expression of GFP in vector control and GFP-tagged β1 transfected cells by Western blotting. (**H**) Expression of GFP in sEVs from WT and GFP-tagged β1 transfected cells by Western blotting. (**I**) Uptake of ExoTracker-labeled sEVs from WT and Δ4–6 mutant cells in MCF7 cells by flow cytometry. (**J**,**K**) Migratory ability of MDA-MB-231 (**J**) and MCF7 (**K**) cells treated with sEVs from WT and mutant cells. Results are presented as mean ± SD from three independent experiments. *** *p* < 0.001. (**L**) Clone formation of MCF7 cells treated with sEVs from WT and mutant cells. ns, not significant. Results are presented as mean ± SD from three independent experiments. Full-length blots/gels of Figure 4B is shown as Figure S3, and Figure 4F–H are shown as Figure S4.

Previous study revealed that sEVs in soluble compounds from MDA-MB-231 cells enhance the migratory ability of MCF7 cells by addition of GW4869, an N-sMase2 inhibitor that blocks ceramide-mediated release of sEVs [1]. Integrin β1 was documented to be present on sEVs and transferred to recipient cells, resulting in enhanced cell migration [1,20], suggesting that integrin β1 might affect the cell migration and adhesion of recipient cells via sEVs. Given the important role of N-glycosylation in the I-like domain of integrin β1, we focused on the Δ4–6 mutant in the subsequent experiments. sEVs from integrin β1 mutants were isolated by differential centrifugation (Figure 4A), in which sEV markers, including CD63, Alix, and TSG101 (Figure 4B), were clearly expressed. sEVs displayed a sphere-like morphology, as evaluated by a transmission electron microscope (Figure 4C), and the sizes of these sEVs were mostly around 100 nm (Figure 4C,D). Not surprisingly, differences in N-glycosylation had no effect on the secretion (Figure 4E) or morphology of sEVs (Figure 4C).

Notably, the presence of integrin β1 in sEVs (termed vesicular β1) from WT-β1 cells and the Δ4–6 mutant (termed WT-β1-sEVs and Δ4–6-sEVs) was clearly revealed by Western blot (Figure 4F). To further verify the presence of the integrin β1 mutant on sEVs, green fluorescent protein (GFP)-tagged integrin β1 was introduced into MDA-MB-231 cells (termed GFP-β1 cells) [21] (Figure 4G). Western blotting showed that GFP was detected on sEVs (Figure 4H), indicating that WT and Δ4–6 mutant β1 could also be packed into sEVs. Flow cytometry analysis revealed that ExoTracker-labeled WT-β1-sEVs and Δ4–6-sEVs were both efficiently taken up by MCF7 cells (Figure 4I), indicating that transfer of vesicular β1 was not affected by N-glycosylation site mutants. To explore the functional role of N-glycosylation differences in vesicular β1, a transwell assay was performed. We found that the migratory ability of both recipient MDA-MB-231 and MCF7 cells was significantly suppressed by Δ4–6-sEVs (Figure 4J,K). Proliferation changes were checked using a colony formation assay, and the colony formation of MCF7 was not altered by Δ4–6-sEVs (Figure 4L).

Collectively, these data reveal that vesicular β1 could be transferred into recipient cells, and N-glycosylation in the I-like domain of vesicular β1 was essential for sEV-mediated metastasis but not proliferation of recipient cells.

## 3. Discussion

Integrin β1 plays a crucial role in cell adhesion, survival, and differentiation [22]. Notably, N-glycosylation in the I-like and membrane-proximal domains of integrin β1 is critical for integrin-mediated functions [14,23]. Integrin β1 is reported to regulate the migratory ability of recipient cells via sEVs [1,7]. We hypothesized that site-specific N-glycosylation could modulate behaviors of recipient cells via transfer of vesicular integrin β1. In the present study, we validated that removal of N-glycosylation in the I-like domain of integrin β1 suppressed cell adhesion and migration via inhibition of FAK phosphorylation. Integrin β1 was present on sEVs and could be transferred into recipient cells. N-glycosylation in the I-like domain of vesicular β1 was found to be essential for sEV-mediated metastasis of recipient cells.

Integrin β1 expression was elevated in constructed mutants compared with cells transfected with an empty vector due to transfection with integrin β1. Despite this, removal of N-glycosylation in different domains of integrin β1 did not alter the molecular weight of β1. This result seems to be inconsistent with a previous study that showed that Δ4–6 and Δ9–12 β1 mutants displayed lower molecular weight [13,14]. This discrepancy might result from the presence of endogenous integrin β1 in mutants in our study, which makes the small molecular weight shift indistinct. Though endogenous integrin β1 still existed in mutants, mutated integrin β1 was highly overexpressed. Constructed mutant cells exhibited similar β1 expression, suggesting that N-glycosylation of β1 did not alter its expression. Removal of N-glycosylation in the I-like domain of integrin β1 was reported to reduce its expression in GE11 cells [14]. The inconsistency may be explained by the fact that inhibition of the main α5β1 heterodimer formation in the Δ4–6 mutant reduced β1 expression in GE11 cells, while the formation of other heterodimers, including the α3β1 dimer, was not affected in the Δ4–6 mutant of MDA-MB-231 cells [14].

We also validated that the presence of integrin β1 in sEVs and vesicular β1 could be transferred to recipient cells. These results are consistent with those of a previous study [1,24]. Vesicular integrin α6β1 could bind lung-resident fibroblasts and epithelial cells governing lung tropism [7]. However, Jeppsen et al. showed that integrin β1 was barely detected in CD81 and CD63-positive sEVs in DKO-1 and Gli36 cells but indicated the presence of integrin β1 in 300–900 nm microvesicles [25]. The discrepancy might result from the diversity of sEVs from different cell lines. Studies have revealed that vesicular cargos can transport functional biomolecules to recipient cells, modulating the cell behavior. For example, exosomal Wnt10b from p85-deficient fibroblasts can promote cancer progression via epithelial-to-mesenchymal transition (EMT) induced by the canonical Wnt pathway [26]. Integrin beta-like 1 (ITGBL1)-enriched EVs from primary colorectal cancer cells were released to activate fibroblasts, resulting in pre-metastatic niche formation and metastatic cancer cell growth [27]. Moreover, sEVs are covered with heavy glycoconjugates, and N-glycosylation plays an essential role in vesicular biological functions. Especially, bisecting GlcNAc modification of vesicular β1 diminishes the sEV-mediated migratory ability of recipient cells [1]. However, biological functions of site-specific N-glycosylation on vesicular β1 is not well-studied. We demonstrated that removal of N-glycosylation in the I-like domain of integrin β1 downregulated cell adhesion and migration of recipient cells. These results indicate that N-glycan, as an informative cargo, has an important role in sEVs and has an influence on behaviors of recipient cells via sEVs.

## 4. Materials and Methods

### 4.1. Cell Culture

Human breast cancer cell lines MDA-MB-231 and MCF7 were obtained from the Cell Bank at the Chinese Academy of Sciences (Shanghai, China). Cells were cultured in Dulbecco’s modified Eagle’s medium (DMEM, Biological Industries, Beit Haemek, Israel) supplemented with 10% fetal bovine serum (FBS, Biological Industries) and 1% penicillin/streptomycin (Beyotime, Haimen, Jiangsu, China) in a humidified incubator at 37 °C and 5% CO_2_ atmosphere.

### 4.2. Plasmid Construction

Human full-length *integrin β1* was amplified from cDNA of MDA-MB-231 cells. Site-directed mutagenesis was performed using fusion PCR. The primer sequences are listed below.

The constructed DNA sequences were cloned into pLVX-AcGFP-N1 plasmids (Takara; Shiga, Japan).

### 4.3. Stable Transfection of WT or Mutant Integrin β1

Empty WT and mutant integrin β1/pLVX-AcGFP-N1 plasmids and ecto-tag-integrin β1 plasmid (provided by D.A. Calderwood, Yale University) were transfected into MDA-MB-231 cells using a lentiviral system as previously described [1,28]. Briefly, constructed lentiviral vectors, along with pMD2.G and psPAX2 (Addgene; Cambridge, MA, USA), were packed into HEK293T cells. Packaged lentiviral particles were collected from the medium supernatant. Then, 2 mL of lentivirus mixture was added to MDA-MB-231 cells in 6-well plates. After 24 h of incubation, the medium was changed, and MDA-MB-231 cells were incubated for an additional 24 h. Stable transfected cells were selected by adding puromycin and confirmed by Western blotting.

### 4.4. Whole-Cell Lysate Extraction and Western Blotting 

Cells were lysed for 30 min at 4 °C with radio-immunoprecipitation assay (RIPA) buffer (50 mM Tris, pH 7.2, 1% Triton X-100, 0.5% sodium deoxycholate, 0.1% SDS, 150 mM NaCl, 10 mM MgCl_2_, 5% glycerol) containing 1% protease inhibitor (Sigma-Aldrich; St. Louis, MO, USA). Lysate was centrifuged at 14,000× *g* for 15 min at 4 °C. The supernatant was collected and the protein concentration was determined with a BCA assay (Beyotime).

For Western blot, proteins (30 μg for total cell proteins; 10 μg for sEV proteins) were separated with SDS-PAGE, and then the gel was transferred onto polyvinylidene difluoride (PVDF) membranes (Bio-Rad; Hercules, CA, USA). The membranes were blocked with 3% (*w*/*v*) bovine serum albumin (BSA, Beyotime) in TBST for 30 min at 37 °C, and probed with primary antibodies (1:1000, *v/v*) against GFP (sc-9996, Santa Cruz Biotechnology, CA, USA); FAK (610087), pFAK (611722) (BD Biosciences, San Jose, CA, USA); Alix (2171s), Calnexin (2433s) (Cell signaling Technology, Danvers, MA, USA); GAPDH (G9545, Merck, Darmstadt, Germany); and integrin β1 (ab30388), TSG101 (ab83), CD63 (ab134045) (Abcam, Cambridge, UK) overnight at 4 °C and incubated with an appropriate horse radish peroxidase (HRP)-conjugated secondary antibody (1:5000, *v/v*) for 30 min at 37 °C. Bands were visualized using enhanced chemiluminescence (Vazyme Biotech, Nanjing, China).

### 4.5. Cell Apoptosis Assay

Cells were detached with trypsin, centrifuged at 500× *g* for 5 min, rinsed with phosphate buffer saline (PBS), and resuspended with 100 μL 1 × binding buffer (BioLegend, San Diego, CA, USA) containing 2.5 μL of APC-Annexin V and 2.5 μL of 7-AAD (BioLegend). Cells were incubated for 20 min in the dark and analyzed by flow cytometry (ACEA Biosciences; San Diego, CA, USA).

### 4.6. Cell Adhesion

Twenty-four-well plates were coated with fibronectin (20 μg/mL; #40105ES08; Yeasen Biotech; Shanghai, China) in PBS overnight at 4 °C and then blocked with 1% BSA for 1 h at 37 °C. Cells were detached, resuspended in serum-free DMEM with 0.1% BSA, and plated onto the fibronectin-coated wells. After 60 min of incubation for MDA-MB-231 cells (4 h for MCF7 cells), non-attached cells were removed by washing with PBS. Attached cells were fixed with 4% paraformaldehyde (Sigma-Aldrich), and representative images were captured by optical microscopy (Sunny optical technology, Zhejiang, China). Cells attached to the fibronectin were counted, and those of triplicate experiments were averaged to give a mean cell count. All the experiments were performed in three duplicates, and results are expressed as mean ± SD of three independent experiments.

### 4.7. Cell Migration by Transwell Assay

Cells were starved with serum-free DMEM for 12 h, then trypsinized and resuspended with serum-free DMEM. Cells (1.5 × 10^4^ cells for MDA-MB-231; 1 × 10^5^ cells for MCF7) in 100 μL of serum-free DMEM were added into the upper chamber of inserts, and complete medium was added to the bottom chamber. After 24 h of incubation, cells on the upper side were removed, and the cells that had migrated across the membrane were fixed with 4% paraformaldehyde, stained with 0.1% crystal violet for 30 min, and photographed with a microscope (magnification 100×). Migrated cells were measured and counted by Image Pro Plus software (Media Cybernetics, Rockville, MD, USA), and those of triplicate experiments were averaged to give a mean cell count for each experiment. All the experiments were performed in three duplicates and results are expressed as mean ± SD of three independent experiments.

### 4.8. Conditioned Medium Extraction, sEV Isolation, and sEV-Free FBS Preparation

Conditioned medium (CM) was prepared as previously described [29]. Briefly, cells were cultured in medium complemented with 10% sEV-free fetal bovine serum for 48 h. The supernatant was collected and centrifuged at 10,000× *g* for 10 min to remove cell debris, and then filtered with a 0.22 μm filter.

To prepare sEVs, cell culture supernatant was sequentially centrifuged at 500× *g* for 10 min, 2000× *g* for 20 min, 10,000× *g* for 30 min at 4 °C, and then ultracentrifuged twice at 100,000× *g* for 70 min. The pellets were resuspended in PBS.

sEV-free FBS was prepared as previously described [30]. Briefly, DMEM medium supplemented with 20% FBS was ultracentrifuged overnight (12 h) at 100,000× *g* and 4 °C. The supernatant was filtered with a 0.22 filter to sterilize it. FBS-free DMEM medium was added to dilute the FBS concentration to 10%, then supplemented 1% penicillin/streptomycin.

### 4.9. Co-Culture System

Recipient cells (1 × 10^5^) were seeded on the bottom chamber and cultured in DMEM supplemented with 10% sEV-free FBS overnight, and donor cells (1 × 10^5^) were seeded on the upper chamber of a 0.4 μm membrane. After 4 days of incubation, recipient cells were collected for phenotype determination.

### 4.10. Nanoparticle Tracking Analysis

sEVs were loaded into a NanoSight LM10 instrument (Malvern; UK), and particles were tracked for 60 s using the NanoSight nanoparticle tracking analysis software program.

### 4.11. Transmission Electron Microscopy (TEM)

Purified sEVs were applied to carbon-coated mesh grids (Electron Microscopy Sciences; Fort Washington, PA, USA) for 5 min, washed with PBS, and stained with 2% freshly prepared uranyl acetate for 30 s [31]. Photos were obtained by TEM (model H-7650; Hitachi; Tokyo, Japan) at 80 kV.

### 4.12. sEV Uptake by Flow Cytometry

sEVs were labeled with an ExoTracker probe as described previously [32]. In brief, sEVs were incubated with ExoTracker (1:500) for 40 min at room temperature, then centrifuged three times with a 10 KD ultrafiltration membrane for 5 min at 14,000× *g* to remove the unlabeled probe. MCF7 cells were treated with labeled sEVs for 1 h and analyzed by flow cytometry (ACEA Biosciences).

### 4.13. Data Analysis

All experiments were reproduced at least three times. Data were statistically analyzed using the GraphPad Prism (GraphPad software; San Diego, CA, USA). Differences between means were evaluated by Student’s *t*-test, and *p*-values < 0.05 were considered significant.

## 5. Conclusions

In summary, removal of N-glycosylation in the I-like domain of integrin β1 reduced β1-mediated activation of FAK, cell adhesion, and migration in MDA-MB-231 cells. Integrin β1 mutants were present on sEVs and could be transferred to recipient cells via sEVs. N-glycosylation in the I-like domain of vesicular β1 is critical for integrin-β1-mediated cell adhesion and migration. Our results may have the potential to modulate the biological function of sEVs and provide a useful basis for further studies of physiological functions of sEVs.

## Figures and Tables

**Table 1:** Primer sequences for PCR.

Oligonucleotide (Integrin β1)	Primer Sequence (5′-3′)	
Full length-sense:	CCGGAATTCATGAATTTACAACCAATTTTCTGGATTGGACT	
Full length-antisense:	GCTCTAGATCATTTTCCCTCATACTTCGGATTGAC	
Δ1–3 mutant	50-sense:	TGTTGAAT*C*TGTGCACCACCCACAATTTGG
	50-anti-sense	TGGTGCACA*G*ATTCAACATTTTTACAGGAAGG
	94 and 97-sense:	GCTACGGT*C*GGTTACAT*C*TTTATTTTTCTTTAT
	94 and 97-anti-sense:	AATAAA*G*ATGTAACC*G*ACCGTAGCAAAGGAAC
Δ4–6 mutant	212-sense:	CTGGTGCAGT*C*CTGTTCACTTGTGC
	212-anti-sense:	TGAAC AG*G*AC TGCAC CAGCC CATT
	269-sense:	TGTAACAT*C*CCTCCAGCCAATCAGTG
	269-anti-sense:	TGGAGG*G*ATGTTACACGGCTGC
	363-sense:	CATTGCTAGAAT*C*TGCAGATAATGTTCC
	363-anti-sense:	TATCTGCA*G*ATTCTAGCAATGTAATTCAGTT
Δ7–8 mutant	406-sense:	TTCCAT*C*CACCCCGTTCTTGCAGTAAG
	406-anti-sense:	CGGGGTG*G*ATGGAACAGGGGAAAAT
	417-sense:	GGAAATAT*C*GGAACATTTTCTTCC
	417-anti-sense:	TGTTCC*G*ATATTTCCATTGGAGATGAGG
Δ9–12 mutant primer	481-sense:	AATGTCCCAT*C*TCCTTCATGACACTT
	481-anti-sense:	CATGAAGGA*G*ATGGGACATTTGAGTG
	520-sense:	CTGAACTGT*C*TTCTTTCCTGCAGTAAGC
	520-anti-sense:	GGAAAGAA*G*ACAGTTCAGAAATCT
	584-sense:	GTGTAGT*C*GGGGTTGCACTCACAC
	584-anti-sense:	GCAACCCC*G*ACTACACTGGC
	669-sense:	CTACCTTGGTAATGT*C*AAAATAGGAACATTC
	669-anti-sense:	CCTATTTT*G*ACATTACCAAGGTAGAAAGT

Note: Bold and italic letters underlined are designed mutations.

## Data Availability

The data used in this study are available from the corresponding author on reasonable request.

## Supplementary Materials

The following are available online at https://www.mdpi.com/1422-0067/22/4/1770/s1, Figure S1: Full-length blots/gels of Figure 2A,F; Figure S2: Full-length blots/gels of Figure 3H,I; Figure S3: Full-length blots/gels of Figure 4B; Figure S4: Full-length blots/gels of Figure 4F–H.

## Abbreviations

sEVs	Small extracellular vesicles
ECM	Extracellular matrix
FAK	Focal adhesion kinase
AKT	protein kinase B
PSI	Plexin-semaphorin-integrin domain
I-EGF	Integrin-epidermal growth factor domain
FN	Fibronectin
CM	Conditioned medium
GFP	green fluorescent protein
FBS	fetal bovine serum
BSA	bovine serum albumin
PBS	phosphate buffer saline
