# Supplementary material for: Role of Site-Specific Glycosylation in the I-Like Domain of Integrin β1 in Small Extracellular Vesicle-Mediated Malignant Behavior and FAK Activation
Source: Int J Mol Sci. 2021 Feb 10;22(4):1770. doi: 10.3390/ijms22041770 (PMC7916680; doi:10.3390/ijms22041770)
Supplement: Supplementary file 1 [file ijms-22-01770-s001.pdf]

Figure 2A

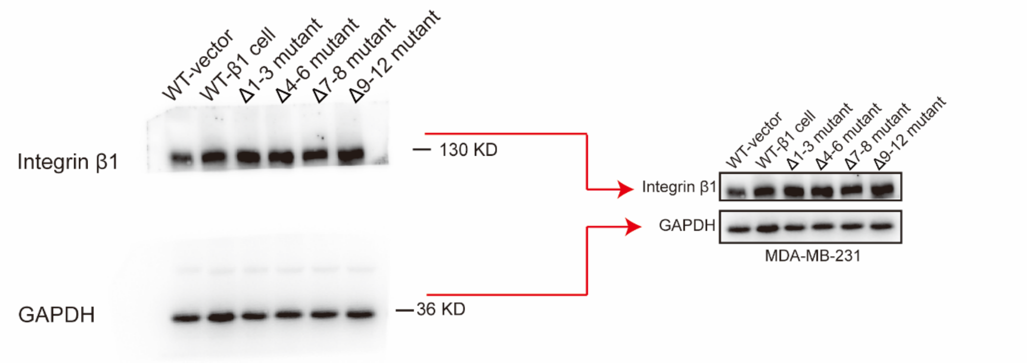


Figure 2F

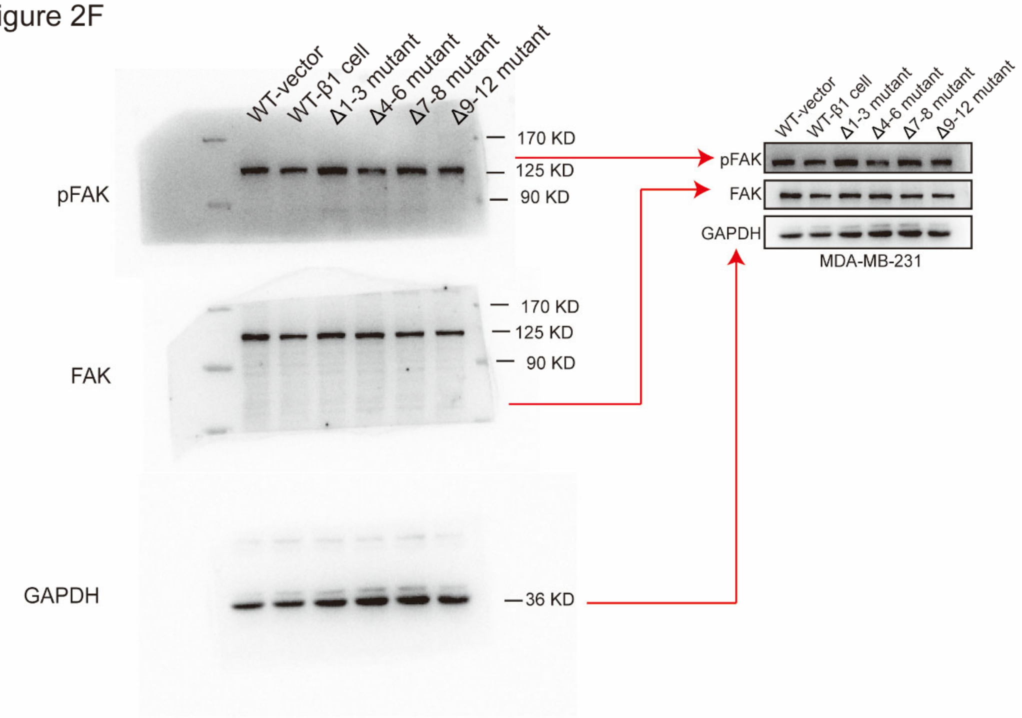


**Supplementary Figure 1**. Full-length blots/gels of Figure 2A, F.

## Figure 3H

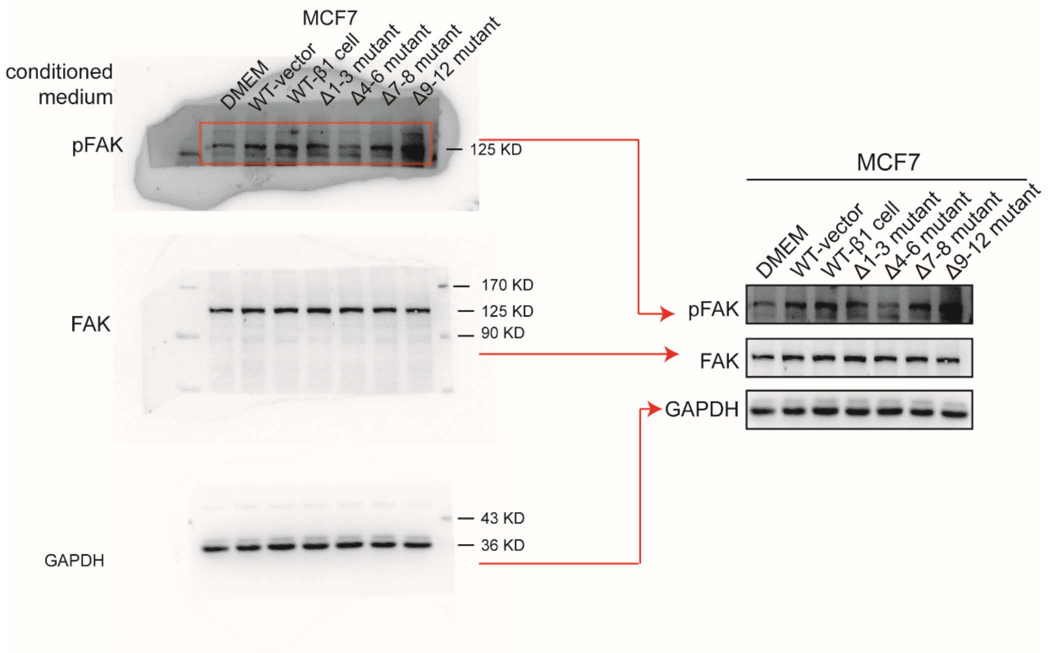


## Figure 3I

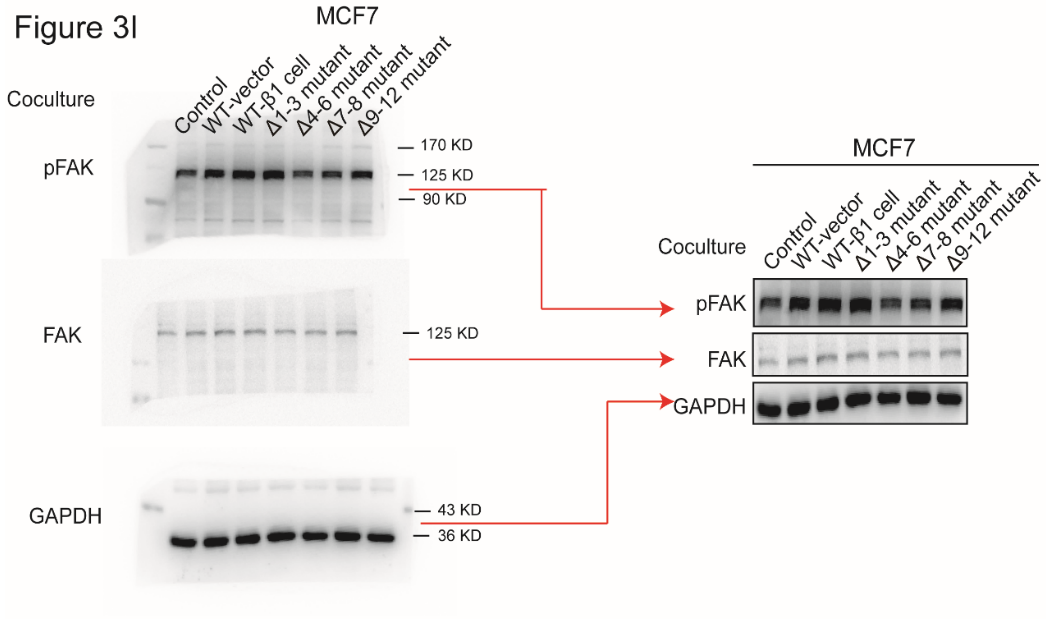


**Supplementary Figure 2**. Full-length blots/gels of Figure 3H, I.

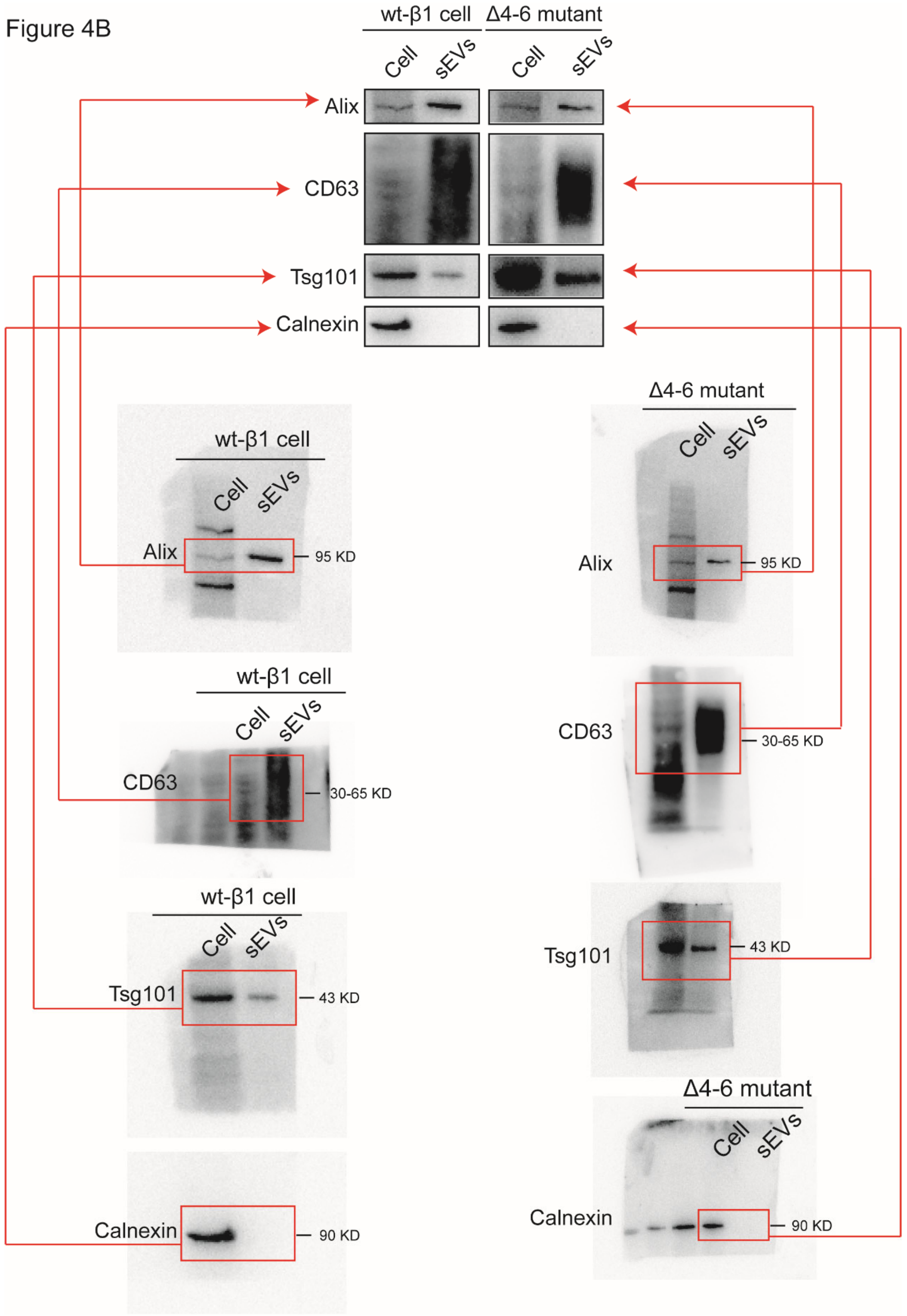


**Supplementary Figure 3**. Full-length blots/gels of figure 4B.

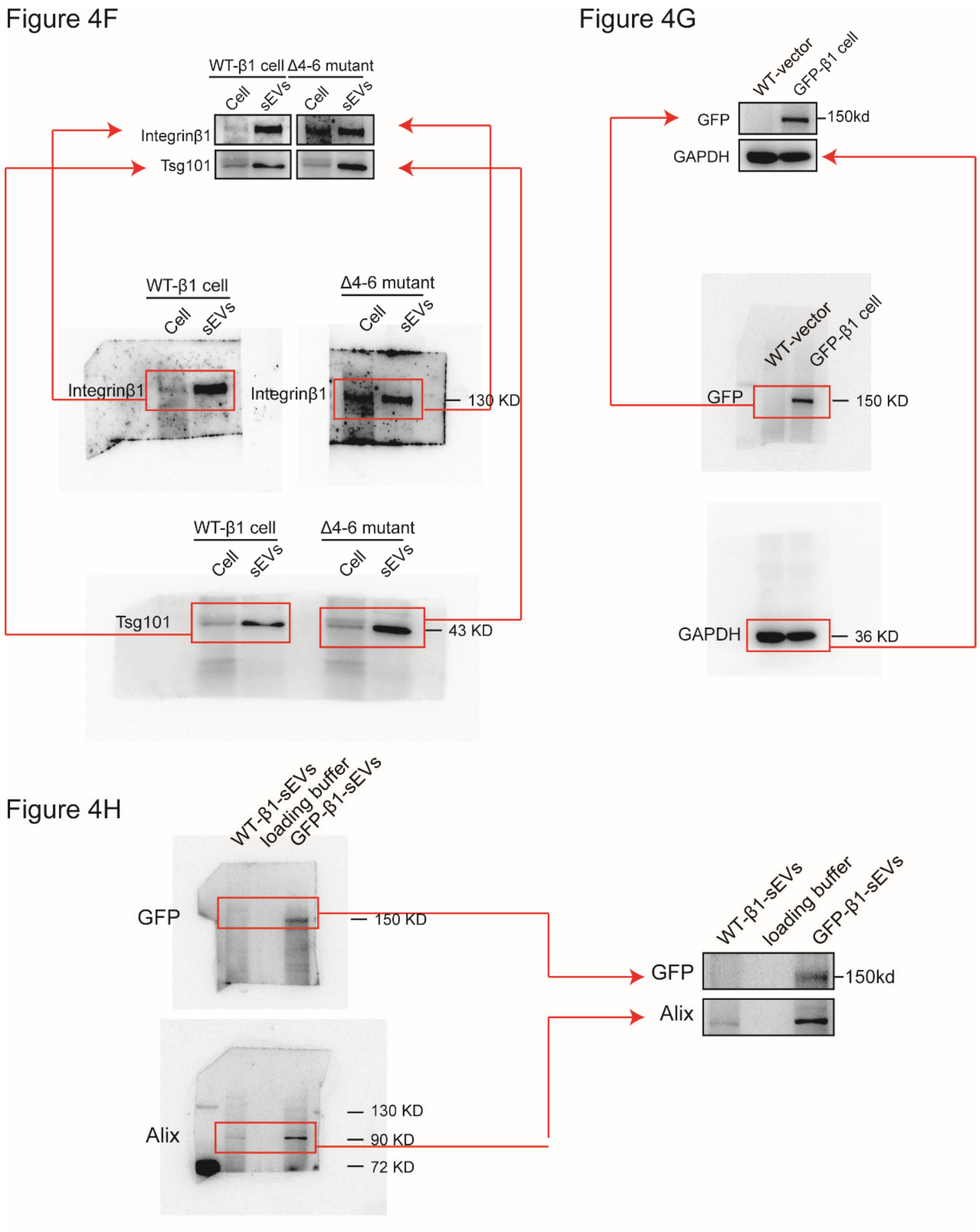


**Supplementary Figure 4.** Full-length blots/gels of Figure 4F, G, H.
